# Feasibility and Safety of Single-Probe Cryoablation with Liquid Nitrogen: An Initial Experience in 24 Various Tumor Lesions

**DOI:** 10.3390/cancers14215432

**Published:** 2022-11-04

**Authors:** Tarek Kammoun, Elodie Prévot, Chris Serrand, Romain Perolat, Hélène de Forges, Nadine Houédé, Jean-Paul Beregi, Julien Frandon

**Affiliations:** 1 Department of Medical Imaging, Nimes University Hospital, University of Montpellier, Medical Imaging Group Nimes, 30029 Nimes, France; 2 Department of Biostatistics, Clinical Epidemiology, Public Health, and Innovation in Methodology (BESPIM), CHU Nimes, 30029 Nimes, France; 3 Gard Cancer Institute, Nimes University Hospital, University of Montpellier, 30029 Nimes, France

**Keywords:** cryoablation, liquid nitrogen, single probe, percutaneous ablation

## Abstract

**Simple Summary:**

Percutaneous cryoablation was developed to minimally perform multi-organ tumor ablations. The most widely known cryotherapy systems use argon gas (high-pressure) and multiple needles to achieve sufficient ablations. The aim of our retrospective study was to assess the feasibility of a new cryotherapy system using single-probe liquid nitrogen for tumor lesions of various sizes and locations, and to evaluate the safety of cryoablation with this technique. Correlations between ice ball sizes and ablation zone sizes with two needle sizes (10G or 13G) and with the freezing duration were evaluated, as well as the sphericity of the ice ball and ablation zones. We showed that this technique is safe in all organs tested. We showed a correlation between the freezing duration and the ice ball size, but not with the ablation zone, which is useful for planning the procedure and treatment conducted by the oncology team.

**Abstract:**

Background: Percutaneous cryoablation with liquid nitrogen is a new technique being used in the treatment of some malignant tumors. Our objective was to assess its feasibility in the ablation of tumor lesions of various sizes and locations. Methods: This retrospective, monocentric study included all consecutive patients who underwent percutaneous cryoablation with liquid nitrogen between December 2019 and March 2021. Cryoablation was performed using 10G or 13G cryoprobes. The ablation volume was measured on post-treatment CT or MRI. Results: 22 patients (24 lesions) were included, 16 of whom were men (73%), while median age was 66 years. The lesions were located in the bone (42%), kidney (29%), soft tissue (17%), lung (8%), or liver (4%). It was feasible in all tumor locations and produced median ablation zones 25 mm in width and 35 mm in length, with a 23 min median freezing time. Freezing duration was correlated with the ice volume (*p* Spearman = 0.02), but not with the ablation volume (*p* = 0.11). The average difference between the ablation zone and ice ball sizes were −6.4 mm in width and −7.7 mm in length. Both ice and ablation volumes were larger when using the 10G probe as compared to when the 13G was used. No complications were reported. Discussion: We showed that this technique was safe and feasible in all organs tested. The freezing duration was correlated with the ice ball size, but not with the ablation zone.

## 1. Introduction

Due to recent advances and the widespread use of computed tomography (CT) and magnetic resonance imaging (MRI), primitive and metastatic tumors are more frequently detected and treated. Interventional radiology is now often considered as the 4th pillar of oncological treatments after chemotherapy, surgery, and radiotherapy [1]. Among the arsenal of potential treatments in interventional radiology, cryotherapy is a cold ablation technique [2]. 

Percutaneous cryoablation with argon gas has been used for its analgesic effect and in the treatment of malignant tumors of the liver, prostate, kidney, and lungs [3,4,5,6,7,8]. However, the use of argon gas, a high-pressure gas, requires a room that is specifically equipped with approved safety mechanisms (300 bar large gas cylinders, validation of the equipment by the institution’s security department), which restricts its general use.

With liquid nitrogen, however, the device used by the technique is mobile, does not have to take place in a specific room, is space-saving, and allows cryoablation to be performed in standard operating rooms. 

A new cryotherapy device using liquid nitrogen (IceSense 3, IceCure Medical Ltd., Caesarea, Israel) has been reported to provide a large ablation zone that requires only one cryoprobe [9], which may reduce the risk of complications, whereas two to four cryoprobes are needed for the argon cryotherapy technique. Another advantage of this new cryotherapy technique is its low cost and easy management. 

While the use of the device has been reported in the treatment of breast fibro-adenoma [10] and some malignant tumors (lung) [11], its feasibility in the treatment of malignant tumors of various locations and heterogeneous tumor stages has not yet been reported.

The objective of this study was to assess the feasibility of this new cryotherapy device that uses liquid nitrogen in the treatment of tumor lesions of various sizes and locations. The secondary objectives were to evaluate the safety of cryoablation—complications related to cryoablation during the procedure and in the following 24 h—and to assess the ablation volume after cryoablation treatment. 

## 2. Patients and Methods

### 2.1. Study Design and Patients

This retrospective, monocentric study was approved by our institutional review board. All consecutive patients who underwent percutaneous cryoablation with the liquid nitrogen ablation device between December 2019 and March 2021 at our institute were included in the study. The exclusion criteria were multiple impacts for a given lesion, prior cryoablation of the zone, concomitant treatment (embolization, radiofrequency), and missing imaging data for the ablation zone measurement.

### 2.2. Cryoablation Technique

Percutaneous cryoablation was validated in a multidisciplinary meeting and was performed for either a palliative, analgesic purpose, for tumor reduction, or with curative intent. Percutaneous cryoablation was performed with a liquid nitrogen ablation device (IceSense 3, IceCure Medical Ltd.) using a single cryoprobe. Two shapes and diameters were available for each cryoprobe: 10-gauge (10G) and 13-gauge (13G) cryoprobes, both producing either elliptical or spherical ablation zones. The ablations were performed by five senior radiologists with more than 5 years of experience in ablation therapies. No specific training was required from the radiologists to be able to use this specific cryotherapy system. Only an explanation of the liquid nitrogen system was given. Cryoablation was performed under local anesthesia, with a single cryoprobe introduced into the tumor with or without the co-axial technique, under ultrasound guidance for soft tissue or liver lesions, or under scanner guidance for other locations. When necessary, the adjacent organs were isolated from the ice ball and protected by the injection of saline or carbon dioxide. The choice of the type of probe and the use of protective techniques were left to the discretion of the operator. Two freezing cycles were performed, with a passive thawing duration between each cycle and an active thawing at the end of the procedure. For lung lesions, three freezing cycles were performed. The ice ball volume was measured at the end of the last freezing cycle on US or CT. The ablation zone volume was measured on post-treatment CT or MR imaging. The sphericity index was calculated (small axis^2^/long axis^2^). The radiation dose was noted at the end of the procedure when performed under CT guidance. Complications related to the cryoablation procedure were collected during the intervention and within the following 24 h.

### 2.3. Anatomopathologic Data

The patients underwent percutaneous biopsy before cryoablation, performed during a single procedure and according to local clinical practices. Data were retrospectively collected from the patients’ files.

### 2.4. Oncologic Follow-Up

Follow-up data were retrospectively assessed at 1 year from preparation of the patients’ files. Secondary curative treatments such as second percutaneous treatment, radiotherapy, and surgery were also reported. 

### 2.5. Statistical Analysis

Considering the retrospective and exploratory character of the study, there was no formal calculation of the patient sample size. All consecutive patients that met the inclusion criteria during the study period were included. The statistical analysis was performed using Biostatgv (http://marne.u707.jussieu.fr/biostatgv, accessed on 29 November 2017) and the R 4.1.1 software (R Core Team (2021). R: A language and environment for statistical computing. R Foundation for Statistical Computing, Vienna, Austria. URL: https://www.R-project.org/, accessed on 8 October 2021). Qualitative variables were described using numbers and proportions, and quantitative variables were represented by medians and ranges. Values were compared using the Wilcoxon–Mann–Whitney test. The Spearman correlation test was used to assess the correlation between the ice ball size and the total duration of the freezing cycles. The ice ball size and the size of the ablation zone were compared, taking the ice ball as reference. Statistical significance was set at *p* < 0.05. 

## 3. Results

### 3.1. Patients

**Figure 1:**
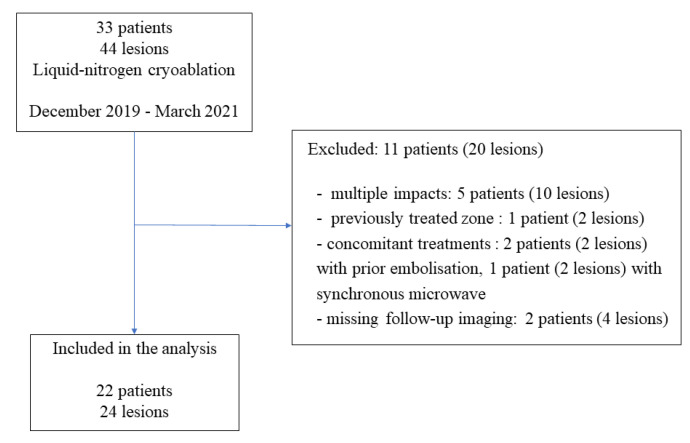
Flow diagram of the study.

Between December 2019 and March 2021, 33 patients underwent percutaneous cryoablation at our institute. Among them, 11 patients (20 lesions) were not included in the study for the following reasons: multiple impact (5 patients), cryoablation of previously treated zone (1 patient), concomitant treatment (3 patients), and missing imaging data (2 patients) (Figure 1). Finally, 22 consecutive patients (24 lesions) were included in the study. The patients were 16 men (73%) and 6 women (27%), and median age was 66 years (range: 36–89). The lesions were located in the bone (42%), kidney (29%), soft tissue (17%), including lymph nodes, lung (8%), and the liver (4%) (Table 1). The median tumor size was 20.5 mm (major axis) (range: 5–38) and 17 mm (minor axis) (range: 5–36) (Table 2). 

### 3.2. Cryotherapy Data

Sixteen (67%) lesions were treated with the 10G cryoprobe and 8 (33%) lesions with the 13G cryoprobe. Four (17%) of the ablations performed with cryoprobes produced a spherical shape, and 20 (83%) ablations produced an elliptical shape. Twenty-one (88%) lesions were treated with two cycles of freezing, and three (13%) lesions (lungs) were treated with three cycles (Table 2). 

The median freezing duration was 21 min (range: 3–42). The median ice ball size was 40.5 mm in length (range: 20–60) for the long axis and 30.5 mm (range: 11–44]) for the short axis. Five ice balls were not measured because of the poor visibility of the ice ball within dense bone tissue under CT scan. The ablation zone was measured under early CT or MR follow-up imaging at day 30 (median) (range: 1–60). The median ablation zone size was 34 mm (range: 16–58) for the long axis and 25 mm (range: 9–40) for the short axis. The average difference between the ice ball and ablation zone measurements were −7.7 mm (+/−5.3 mm) for the long axis and −6.4 mm (+/−5.1 mm) for the short axis. 

**Figure 2:**
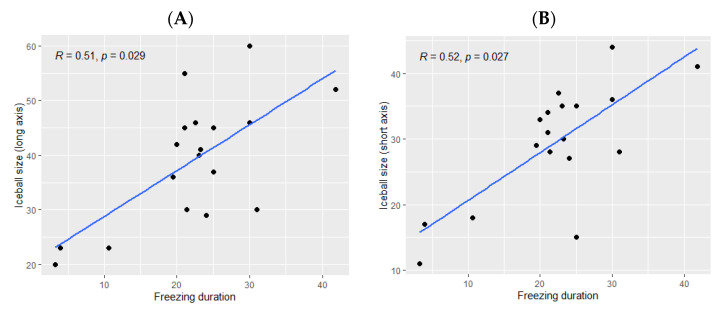
Correlation between the ice ball size and the total freezing duration; (**A**): major axis; (**B**): minor axis. The correlations are represented with Spearman coefficient (R). A coefficient close to 1 shows a strong positive correlation, while a coefficient close to −1 shows a strong negative correlation. The *p*-value (*p*) indicates the significance level of the observed correlation.

**Figure 3:**
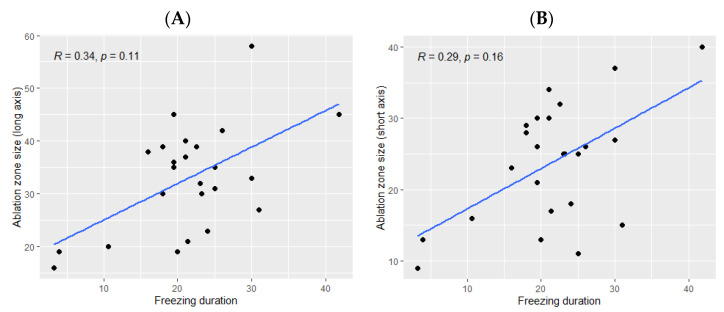
Correlation between the size of the ablation zone and the total freezing duration; (**A**): major axis; (**B**): minor axis. The correlations are represented by the Spearman coefficient (R). A coefficient close to 1 shows a strong positive correlation, while a coefficient close to −1 shows a strong negative correlation. The *p*-value (*p*) indicates the significance level of the observed correlation.

The ice ball size was significantly bigger when the total freezing duration was longer (*p* Spearman = 0.029 for the long axis and *p* = 0.02 for the short axis, Figure 2). However, the freezing duration was not significantly correlated with the ablation zone size (*p* = 0.11 for the long axis and *p* = 0.16 for the short axis, Figure 3).

**Figure 4:**
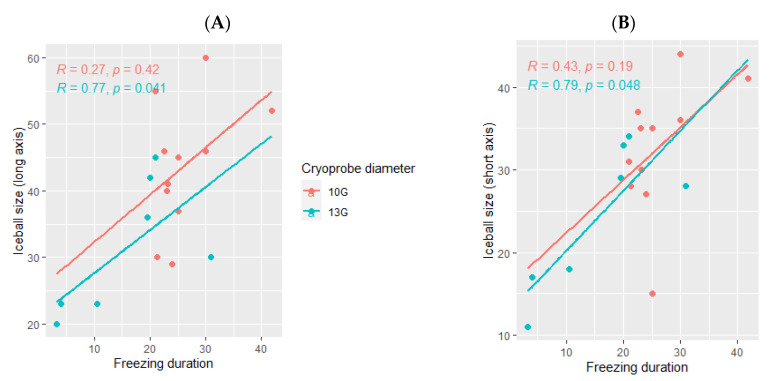
Correlation between the ice ball size and the total freezing duration, depending on the cryoprobe used (10G: orange; 13G: blue); (**A**): major axis; (**B**): minor axis. The correlations are represented by the Spearman coefficient (R). A coefficient close to 1 shows a strong positive correlation, while a coefficient close to −1 shows a strong negative correlation. The *p*-value (*p*) indicates the significance level of the observed correlation.

The ice ball size was larger when using the 10G cryoprobe (45 mm (range: 29–60) for the long axis and 35 mm (range: 15–44) for the short axis), obtained with a median freezing duration of 24 min (range: 18–42) as compared with the size of the ice balls produced using the 13G cryoprobe (30 mm (range: 20–45) for the long axis and 28 mm (range: 11–34) for the short axis), with a median freezing duration of 18 min (range: 3–31). The ice ball size was correlated with the freezing duration only when using the 13G cryoprobe (*p*= 0.041 for the long axis and *p* = 0.048 for the short axis, Figure 4) and not the 10G cryoprobe (*p* = 0.42 for the long axis and *p* =0.19 for the short axis, Figure 4).

**Figure 5:**
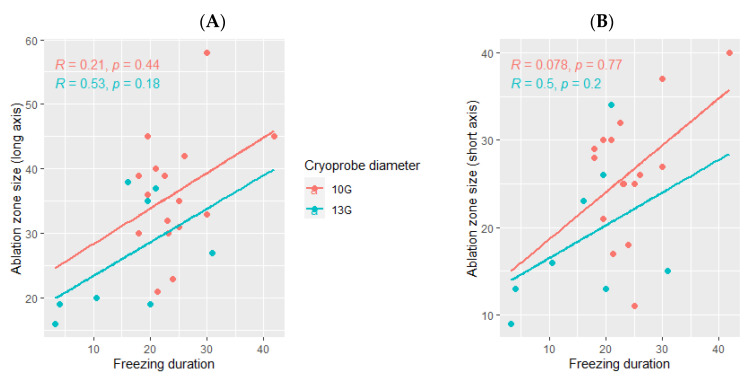
Correlation between the volume ablation and the total freezing duration, depending on the cryoprobe used (10G: orange; 13G: blue); (**A**): major axis; (**B**): minor axis. The correlations are represented by the Spearman coefficient (R). A coefficient close to 1 shows a strong positive correlation, while a coefficient close to −1 shows a strong negative correlation. The *p*-value (*p*) indicates the significance level of the observed correlation.

The median delay between the cryotherapy procedure and the follow-up imaging in which the ablation was measured was 30 days (range: 1–60). The ablation zone was larger using the 10G cryoprobe (35.5 mm (range: 21–58) for the long axis and 26.5 mm (range: 11–40) for the short axis) as compared with the ablation zones obtained using the 13G cryoprobe (23.5 mm (range: 16–38) for the long axis and 15.5 mm (range: 9–34) for the short axis, Figure 5).

The median sphericity index was higher for the ice balls, 0.87 (range: 0.86–0.87) than for the ablation zones, 0.49 (range: 0.30–0.65) for all cryoablations. When using the elliptical cryoprobe, the sphericity index was not significantly different between the ice balls and the ablation zones, whereas it was significantly different when using the spherical cryoprobe. 

### 3.3. Complications

There were no complications reported during the procedures nor in the following 24 h. In particular, no hematoma was reported for all cryoablations, and no pneumothorax was reported for the two pulmonary ablations.

### 3.4. Dosimetry

The median radiation dose for these procedures performed under CT guidance was 1122 mGy.cm (range: 33–4010).

### 3.5. Oncologic Outcome

Some patients underwent secondary curative treatment (*n* = 2 secondary cryotherapy, *n* = 1 radiotherapy) during the year following the procedure (Table 2). During the follow-up after 1 year, 10 patients (45.5%) had achieved a complete response and 6 patients (27.3%) had achieved a local response, but with distant progression (Table 2), i.e., 72.7% local efficacy of the cryoablation treatment at 1 year.

## 4. Discussion

In this retrospective study, we showed that liquid nitrogen percutaneous cryoablation under US or CT guidance is feasible and safe for a wide variety of tumor lesions. This study, which used a new liquid nitrogen cryoablation technique, is the first to be conducted on patients with tumors in various locations. It gives an overview of the ice balls and ablation zones that could be obtained, depending on the total freezing duration and on the size of the cryoprobe used. Our results showed that the ice ball size was correlated with the freezing duration, whereas the ablation zones were smaller than the ice balls, and their sizes were not correlated with the freezing duration. Both the ice balls and ablation zones were larger when using the 10G cryoprobe as compared to when the 13G cryoprobe was used.

The ice ball, corresponding to the visible 0 °C ice front, was measured immediately at the end of the last freezing cycle using US or CT. It was poorly visible in dense bone, as previously described for argon cryotherapy [12]. The ablation zone, which corresponded to the lethal zone (between −19 °C and −40 °C, depending on the tissue [12]), was measured during the follow-up imaging (CT or MRI) [13,14]. We showed that a longer freezing duration was significantly associated with a larger ice ball, but it was not significantly correlated with the ablation zone size. This may be due to tissue retraction, which depends on the delay in the follow-up imaging, as described in the literature for microwave ablation [15]. Another explanation may be the small number of patients and the limited power used in our study.

Our results showed that 10G cryoprobes produced larger ice balls and larger ablation zones than 13G cryoprobes. Overall, ice balls measuring around 4.5 cm in length from the 10G probes and 3 cm from the 13G probes could be obtained with a 20 min total freezing duration. Nomori et al. reported that the diameter of the ice balls in gel using the 13G and 10G cryoprobes reached 3.9 ± 0.1 and 4.8 ± 0.3 cm, respectively, after 15 min of freezing [9]. They also reported ice ball sizes of 4.6 ± 0.3 cm with the 13G cryoprobe and 5.3 ± 0.4 cm with the 10G cryoprobe in ex vivo pig lungs after 3 freezing cycles of 7 min each (total freezing duration of 21 min). The smaller size of our ice balls could be explained by the in vivo conditions of our study as compared to the ex vivo or in-gel conditions in that study, without the cold-sink effect related to blood flow [16].

In our study, the average difference between the ablation zone and the ice ball sizes was −6.4 mm (+/−5.1 mm) for the short axis and −7.7 mm (+/−5.3 mm) for the long axis. This size difference may be explained by the difference between the measurement of the ice ball, i.e., icefront obtained immediately after the procedure, and the ablation zone, i.e., lethal zone measured during the follow-up imaging; the other explanation is the occurrence of tissue retraction between the two measurement times [12,17,18]. These results provide significant information on the non-lethal margin area and highlight the need to oversize the volume of the ice ball, depending on the lesion targeted, in order to avoid tumor recurrence.

It has been shown that the histological damage from cryoablation was greater after two freezing cycles as compared to one freezing cycle, with a microscopically more extensive necrosis zone [19]. We believe that cryoablation protocols should thus include at least two freezing cycles. In our study, protocols of two or three freezing cycles were performed. Three-cycle protocols were chosen for lung lesions, as previously described in the literature [11]. A previous study showed that the thawing process (both active and passive) did not impact tissue damage [19], but more recent studies have demonstrated that active thawing resulted in increased cell survival, and thereby less effective ablation, as compared with passive thawing [20]. In our study, passive thawing was performed.

The size of the ice balls was shown to depend on the “cold-sink effect” [16], i.e., the influence of the vessels near the cryoablation area limiting the growth of the ice ball. The “heat-sink effect” is a well-established phenomenon in radiofrequency ablation, in which tumors adjacent to large vessels are sometimes insufficiently ablated because perfusion-mediated tissue cooling reduces coagulation necrosis by heat energy [21]. An opposite mechanism has been described for cold ablation techniques [22]. In our study, the largest ice ball was obtained in a fatty soft tissue with poor vascularization, whereas the smallest ablation zones were obtained in the treatment of hypervascular lesions (thyroid metastasis). These considerations are useful when choosing the optimal placement of the cryoprobe within the targeted organ as they ensure adequate safety margins [23].

In our study, the sphericity index was calculated according to a previously published formula (short axis^2^/long axis^2^) [15]. It was higher for the ice balls than for the ablation zones, which were more elliptic, probably because tissue contraction may be stronger along the shorter axis than the longer axis. The difference was not significant for ablations performed with the spherical cryoprobe; however, the number of patients was too small. For elliptic probes, a smaller lateral margin may impact oncologic outcome.

Cryoablation was shown to be a safe technique; only a few complications have been reported in the literature, which were mainly hemorrhages or injuries to adjacent organs or structures [24,25]. In our study, no complications were reported. The system was easy to use for the whole team, without specific expertise being required. With cryoablation, the growth of the ice ball, and thus of the ablation zone, can be controlled in real time by ultrasound or CT [26,27], as opposed to other percutaneous treatments such as radiofrequency or microwave therapies. There was no or little damage to the tissue adjacent to the frozen lesion, and when necessary, the adjacent organs could be isolated from the ablation area and protected by the injection of saline or carbon dioxide [28,29]. Finally, the use of a single cryoprobe, which is specific to this liquid nitrogen cryoablation technique, may have contributed to limiting the occurrence of complications commonly reported for most percutaneous ablation techniques.

The primary efficacy of cryotherapy, i.e., the local response, was 77% in this heterogeneous population. These results are lower than those previously published in the literature for a selected population of patients with clear-cell renal carcinoma or low-stage (T1A) pulmonary carcinoma [11,30], although our study included various pathologies or various stages, and thus had poorer prognoses in terms of progression or recurrence. As for other single-probe ablations, a limitation of the technique is the size of the tumor. Partial responses were reported, most often for large (>3 cm) tumors. It is thus important to keep to the percutaneous ablation guidelines [31] and to perform cryoablation with this technique only for tumors <3 cm. For larger tumors, in our view, alternative strategies should be chosen, such as multiple ablations (single-probe ablation in multiple steps, with a repositioning of the probe for each ablation) or the use of a multiple-probe technique.

This study has some limitations, among which is the small number of patients that is concordant with its pilot and feasibility design. It also did not allow for analysis according to the location of the lesion. Furthermore, no correlation was found between the ablation zone and the freezing time, which may be explained by tissue retraction, but also by the lack of power in the study. Finally, correlations related to the sphericity of the ice ball and the ablation volumes, depending on the type of cryoprobe used (elliptic or spheric), were not obtained due to the limited number of patients. It is now essential to conduct larger oncologic efficacy studies to assess cryoablation at each tumor location and to determine optimized ablation protocols according to the analgesic or curative intent. Additionally, this new device using a single-probe procedure and liquid nitrogen is ergonomic and easy-to-use. The duration of the procedure, the learning curve as well as medico-economic results may be interesting to study in the future.

## 5. Conclusions

Percutaneous liquid nitrogen cryotherapy used on kidney, bone, liver, lung, and soft tissue tumors is feasible, producing ablation zones of around 35 mm (long axis) with the use of 10G cryoprobes and 25 mm (long axis) with 13G probes, at around 20 min of freezing. The technique was safe, with no complications reported. Long-term efficacy studies now need to be conducted for each organ and type of lesion, both for tumor response or analgesic purposes, to determine optimized ablation protocols.

## Figures and Tables

**Table 1:** Patient and lesion characteristics.

Patient Characteristics	N = 22
Gender, *n* (%)	
Male	16 (73%)
Female	6 (27%)
Median age (years) (range)	66 (36–89)
**Lesion Data**	**N = 24**
Location, *n* (%)	
Bone (central)	10 (42%)
Kidney	7 (29%)
Soft tissue Lung	4 (17%) 2 (8%)
Liver	1 (4%)
Cryoablation intent, *n* (%)	
Palliative analgesic	8 (33%)
Curative	16 (67%)

**Table 2:** Cryoablation data and oncologic outcomes.

				Ice Ball			Ablation Zone			Difference between the Ice balls and Ablation Zones					
Lesion Location	Tumor Size (Major × Minor Axis, mm)	Cryoprobe Type	Cryoprobe Size	Length (mm)	Width (mm)	Sphericity Index	Major Axis (mm)	Minor Axis (mm)	Sphericity Index	Major Axis (mm)	Minor Axis (mm)	Freezing Duration (min)	Anapathological Results	Associated/ Follow-Up Treatment	Follow-Up at 1 Year
Bone	33 × 33	Elliptic	10G	37	15	0.1	31	11	0.1	−6	−4	25	Metastasis from thyroid cancer	Second cryotherapy	Stable disease
Bone	14 × 14	Elliptic	13G	23	18	0.6	20	16	0.6	−3	−2	10.6	Metastasis from neuroendocrine carcinoma	Radio therapy	Partial response
Bone	15 × 11	Elliptic	13G	23	17	0.5	19	13	0.4	−4	−4	4	Metastasis from breast cancer	Right leg amputation	Stable disease
Bone	10 × 9	Elliptic	13G	Not measurable *			38	23	0.3	Not measurable		16	Metastasis from uterine sarcoma	None	Complete local response Distant progression
Bone	24 × 21	Elliptic	10G	Not measurable *			36	21	0.3	Not measurable		19.5	Metastasis from pulmonary adenocarcinoma	None	Complete response
Bone	16 × 15	Elliptic	10G	Not measurable *			45	30	0.4	Not measurable		19.5	Metastasis from bronchial adenocarcinoma	None	Complete response
Bone	38 × 29	Elliptic	10G	Not measurable *			39	29	0.5	Not measurable		18	Metastasis from colon adenocarcinoma	None	Lost to follow-up
Bone	20 × 20	Spherical	10G	Not measurable *			42	26	0.3	Not measurable		26	Metastasis from clear cell renal carcinoma	None	Complete local response Distant progression
Bone	20 × 15	Elliptic	10G	Not measurable *			30	28	0.8	Not measurable		18	Metastasis from breast cancer	None	Complete response
Bone	28 × 24	Elliptic	10G	46	37	0.6	39	32	0.6	−7	−5	22.5	Metastasis from thyroid cancer	None	Complete local response Distant progression
Kidney	21 × 16	Elliptic	13G	36	29	0.6	35	26	0.5	−1	−3	19.5	Clear cell renal carcinoma	None	Complete response
Kidney	19 × 17	Spherical	10G	29	27	0.8	23	18	0.6	−6	−9	24	Clear cell renal carcinoma	None	Complete response
Kidney	36 × 36	Elliptic	10G	52	41	0.6	45	40	0.8	−7	−1	42	Clear cell renal carcinoma	None	Complete response
Kidney ^1^	21 × 18	Elliptic	10G	45	35	0.6	35	25	0.5	−10	−10	25	Clear cell renal carcinoma	None	Complete response
Kidney ^1^	16 × 16	Spherical	10G	30	28	0.8	21	17	0.6	−9	−9	21.3			
Kidney	27 × 23	Elliptic	10G	46	36	0.6	33	27	0.6	−13	−9	30	Clear cell renal carcinoma	None	Complete response
Kidney	25 × 17	Elliptic	10G	40	35	0.7	32	25	0.6	−8	−10	23	Clear cell renal carcinoma	None	Complete response
Soft tissue	5 × 5	Elliptic	13G	20	11	0.3	16	9	0.3	−4	−2	3.3	Neurinoma	None	Alive without pain (VAS 0/10)
Soft tissue	20 × 10	Elliptic	13G	42	33	0.6	19	13	0.4	−23	−20	20	Metastasis from melanoma	Second cryotherapy	Complete local response Distant progression
Soft tissue	29 × 29	Elliptic	10G	60	44	0.5	58	37	0.4	−2	−7	30	Liposarcoma	None	Complete local response Distant progression
Soft tissue	31 × 22	Elliptic	10G	41	30	0.5	30	25	0.7	−11	−5	23.2	Chondrosarcoma	None	Complete response
Lung ^2^	11 × 11	Elliptic	13G	45	34	0.5	37	34	0.8	−8	0	21	Metastasis from cortico-surrenaloma	None	Complete local response Distant progression
Lung ^2^	15 × 11	Elliptic	10G	55	31	0.3	40	30	0.5	−15	−1	21			
Liver	21 × 19	Spherical	13G	30	28	0.8	27	15	0.3	−3	−13	31	Hepatocellular carcinoma	None	Complete response

* The ice ball was not measurable because it was not visible in dense bone tissue. ^1^ and ^2^: Two patients had two lesions treated with cryoablation.

## Data Availability

Study data may be accessed upon reasonable request to the corresponding author.
